# 单倍型造血干细胞移植前血细胞恢复状态对急性髓系白血病患者预后的影响

**DOI:** 10.3760/cma.j.issn.0253-2727.2021.12.012

**Published:** 2021-12

**Authors:** 晨 赵, 于谦 孙, 兰平 许, 晓辉 张, 开彦 刘, 晓军 黄, 昱 王

**Affiliations:** 1 北京大学人民医院、北京大学血液病研究所、国家血液系统疾病临床医学研究中心、造血干细胞移植北京市重点实验室 100044 Peking University People's Hospital, Peking University Institute of Hematology, National Clinical Research Center for Hematologic Disease, Beijing Key Laboratory of Hematopoietic Stem Cell Transplantation, Beijing 100044, China; 2 北京大学血液学协同创新中心 100044 Hematology Collaborative Innovation Center, Peking University, Beijing 100044, China

急性髓系白血病（AML）约占所有白血病的59％，异基因造血干细胞移植（allo-HSCT）是其有效治疗手段[1]–[5]。除了年龄、疾病分层等影响AML患者造血干细胞移植的预后因素以外，移植前的疾病缓解状态对疾病的预后也有重要影响[6]–[8]，而且被纳入EBMT评分系统的预后分层中[9]。近年来，微小残留病灶（MRD）监测被广泛应用于AML移植的患者[10]–[12]，且研究表明移植前MRD阴性患者的长期生存优于移植前MRD阳性患者，复发率也明显降低[13]–[15]。研究显示，AML患者诱导化疗后获得血细胞未恢复的完全缓解同低生存、高复发率相关[16]–[17]。而在allo-HSCT患者中，移植前血细胞恢复状态对AML患者移植预后的影响也尚存在争议。最近一项研究发现，AML患者移植前血细胞恢复状态对5年OS率无明显影响[18]，另一项allo-HSCT研究中，移植前血细胞未恢复完全缓解的AML患者具有较高的非复发死亡率[19]。在单倍型造血干细胞移植（haplo-HSCT）中尚无相关研究数据。为此，我们回顾性分析180例AML患者的临床资料，探讨移植前血细胞恢复状态对haplo-HSCT预后的影响。

## 病例与方法

1. 病例资料：本研究纳入2018年1月至2018年12月期间在北京大学血液病研究所接受haplo-HSCT的180例AML患者（不含急性早幼粒细胞白血病），移植前均获得血液学完全缓解，包括血细胞恢复的形态学完全缓解（CR）、血小板未恢复的形态学完全缓解（CRp）、血小板和中性粒细胞均未恢复的形态学完全缓解（CRi）。

2. 移植、预处理方案：移植方案见文献[20]。haplo-HSCT常规预处理方案（北京方案）见文献[11], [21]；改良Bu/Cy+ATG方案：阿糖胞苷（Ara-C）4 g/m^2^静脉滴注，−10 d、−9 d；白消安（Bu）0.8 mg/kg静脉滴注每6 h 1次，−8 d～−6 d；环磷酰胺（Cy）1.8 g·m^−2^·d^−1^静脉滴注，−5 d、−4 d；司莫司汀250 mg/m^2^口服，−3 d；抗胸腺细胞球蛋白（ATG）2.5 mg·kg^−1^·d^−1^静脉滴注，−5 d～−2 d。

3. 定义：血液学缓解的定义参见文献[22]。CR：①骨髓原始细胞<0.050且无奥氏小体；②无骨髓外白血病残留证据；③PLT≥100×10^9^/L；④外周血中性粒细胞绝对计数（ANC）≥1×10^9^/L；⑤脱离红细胞输注。CRp：①骨髓原始细胞<0.050；②ANC≥1×10^9^/L；③PLT<100×10^9^/L。CRi：①骨髓原始细胞<0.050；②ANC<1×10^9^/L。中性粒细胞植入：ANC≥0.5×10^9^/L连续3 d；血小板植入：PLT≥20×10^9^/L连续7 d且脱离血小板输注。急性及慢性GVHD诊断标准参照文献[21]。

4. 多参数流式细胞术（MFC）检测MRD：采用8色MFC检测骨髓细胞MRD。骨髓和外周血的评估至少在末次化疗后28 d、预处理前2周内进行。检测MRD的8色抗体组合包括抗CD7、CD11b、CD13、CD14、CD16、CD19、CD33、CD34、CD38、CD41、CD45、CD56、CD61、CD64、CD71、CD117、CD123和HLADR抗体。本组病例所用流式细胞仪型号为FACSCantoⅡ。每管收集（0.2～1.0）×10^6^个细胞，并设立同型对照。MRD阳性定义为检测到白血病相关免疫表型（LAIP）的异常细胞群（至少检测到25个细胞），且至少2个标志与初诊时LAIP相同。检测到异常细胞的比例为占总CD45^+^细胞的比例。质量控制流程参照文献[23]。

5. 随访：随访资料来自电话随访、住院/门诊病历。随访截止日期：2020年12月30日。总生存（OS）时间：移植物末次回输至随访截止或死亡的日期。无白血病生存（LFS）时间：移植物末次回输至随访截止或复发/死亡的日期。

6. 统计学处理：分类变量采用*χ*^2^检验或fisher精确检验，连续变量采用Student's *t*检验或Mann-Whitney *U*检验。采用Kaplan-Meier法绘制生存曲线，OS、LFS率的组间比较采用Log-rank检验。采用Cox模型进行单因素和多因素分析，*P*<0.05的参数纳入Cox 多因素分析。采用SPSS软件进行数据分析。复发、移植相关死亡为竞争风险，采用R软件cmprsk包进行竞争风险生存模型的比较。

## 结果

1. 患者一般资料：180例患者中男101例，女79例，中位年龄32（2～60）岁。根据移植前疾病状态分为CR组（131例）、CRi组（26例）、CRp组（23例），三组之间在性别、移植前形态学缓解次数、细胞/分子遗传学危险分级、供受者关系、供受者血型、回输单个核细胞和CD34^+^细胞数量方面差异均无统计学意义（*P*>0.05），详见表1。

**表1:** 单倍型造血干细胞移植前不同血细胞恢复状态急性髓系白血病患者临床特征比较

特征	CR组（131例）	CRi组（26例）	CRp组（23例）	统计量	*P*值
年龄［岁，*M*（范围）］	31（2～60）	23（2～45）	32（10～55）	3.218	0.271
性别［例（男/女）］	70/61	16/10	15/8	1.466	0.480
移植前疾病状态［例］				2.034	0.362
HCR1	112	21	17		
HCR2	19	5	6		
细胞遗传学/分子遗传学危险分级［例］				6.205	0.184
低危	17	5	2		
中危	87	11	16		
高危	27	10	5		
供受者关系［例］				2.896	0.822
父母	54	13	9		
子女	40	5	7		
同胞	31	7	7		
其他	6	1	0		
供受者血型相合程度［例］				6.887	0.142
全相合	76	17	12		
主要不合	36	2	5		
次要不合	19	7	6		
回输MNC［×10^8^/kg，*M*（范围）］	8.78（6.33～14.90）	7.24（6.14～12.81）	6.98（5.81～11.90）	0.393	0.451
回输CD34^+^细胞［×10^6^/kg，*M*（范围）］	2.13（0.64～7.49）	1.98（0.96～4.49）	2.95（0.68～9.81）	0.313	0.690

注：CR：血细胞恢复的形态学完全缓解；CRp：血小板未恢复的形态学完全缓解；CRi：中性粒细胞和血小板均未恢复的形态学完全缓解；HCR1、HCR2分别为第1、2次形态学完全缓解；MNC：单个核细胞

180例患者均获得粒细胞植入，中位植入时间为13（8～22）d，174例（96.7％）获得血小板植入，中位植入时间为22（8～271）d。全部患者中，125例（69.4％）患者发生急性GVHD，其中Ⅰ/Ⅱ度103例（82.4％），Ⅲ/Ⅳ度22例（17.6％）。38例（21.1％）患者发生慢性GVHD，其中轻、中、重度分别为23、2、11例。117例（65.0％）发生巨细胞病毒（CMV）感染，27例（15.0％）发生EB病毒感染。

中位随访时间为680（50～1414）d，24例（13.3％）患者死亡，其中10例死于复发，14例死于移植相关合并症，移植后2年OS率为（86.8±2.7）％。180例患者中，24例复发（13.3％），中位复发时间为移植后276（30～730）d，移植后2年累积复发率（CIR）为（16.2±0.5）％。复发后治疗：二次移植2例，化疗+供者淋巴细胞回输（DLI）6例，单纯化疗11例，未接受治疗5例。接受二次移植的2例患者中，1例获得CR，另1例二次移植后2个月再次复发死亡。17例接受化疗+DLI或化疗的患者中，13例获得CR，4例死于复发。5例未接受治疗的患者均死于复发。

2. 移植前MRD对移植预后的影响：180例患者中有54例（30.0％）移植前MRD阳性，MRD阳性组和阴性组患者均获得中性粒细胞植入，中位植入时间分别为13（9～21）d、12（8～22）d（*P*＝0.546）。MRD阴性组中124例（98.4％）患者获得血小板植入，中位植入时间为21（8～271）d；MRD阳性组中50例（92.6％）获得血小板植入，中位植入时间为23（8～117）d（*P*＝0.046）。MRD阴性组72例患者发生Ⅰ/Ⅱ度急性GVHD，14例患者发生Ⅲ/Ⅳ度急性GVHD，移植后30 d急性GVHD累积发生率为（68.6±2.5）％；MRD阳性组31例患者发生Ⅰ/Ⅱ度急性GVHD，I8例患者发生Ⅱ～Ⅳ度急性GVHD，移植后30 d急性GVHD累积发生率为（11.6±1.2）％（*P*<0.001）。随访结束，MRD阴性组死亡10例，2年OS率为（91.9±2.5）％，MRD阳性组死亡14例，2年OS率（75.8±6.2）％（*P*＝0.003）。两组2年LFS分别为（84.4±3.5）％，（67.0±6.6）％（*P*＝0.002）。MRD阴性组、阳性组分别有10、14例患者复发，2年CIR分别为（7.5±0.1）％、（25.5±0.3）％（*P*＝0.011）。两组2年TRM分别为（6.5±0.1）％、（9.0±0.1）％（*P*＝0.028）。

3. 移植前血细胞恢复状态对移植预后的影响：全部患者中，CR组131例（72.8％），CRp+CRi组49例（27.2％），两组中性粒细胞全部植入，中位植入时间为13（8～22）d、14（11～21）d（*P*＝0.167）。两组血小板植入分别为127例（96.％）、47例（95.9％），中位植入时间分别为22（8～271）d、24（8～117）d（*P*＝0.290）。CR组患者发生Ⅰ/Ⅱ度急性GVHD 72例，Ⅲ/Ⅳ度急性GVHD 17例，移植后30 d急性GVHD累积发生率为（62.9±2.6）％。CRp+CRi组发生Ⅰ/Ⅱ度急性GVHD 31例，Ⅲ/Ⅳ度急性GVHD 5例，移植后30 d急性GVHD累积发生率为（13.5±1.3）％，低于CR组（*P*<0.001）。两组2年OS率分别为（87.2±3.0）％、（89.2±4.6）％（*P*＝0.826）（图1A）。2年LFS率分别为（76.8±3.9）％、（85.2±5.2）％（*P*＝0.620）（图1B）。CR组、CRp+CRi组分别有20、4例患者复发，移植后2年CIR分别为（16.1±0.1）％、（8.6±0.2）％（*P*＝0.264）。两组2年TRM分别为（7.1±0.1）％，（6.2±0.1）％（*P*＝0.486）。

**图1:**
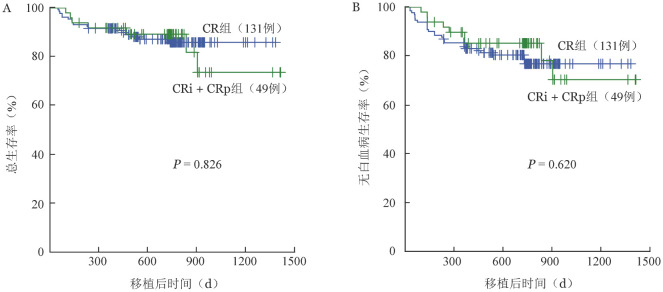
不同血小板恢复状态急性髓系白血病患者单倍型造血干细胞移植后总生存曲线（A）和无白血病生存曲线（B） CR：血细胞恢复的形态学完全缓解；CRp：血小板未恢复的形态学完全缓解；CRi：血小板和中性粒细胞均未恢复的形态学完全缓解

4. 影响移植预后的分析：将年龄、性别、移植前形态学缓解次数、细胞/分子遗传学危险分级、供受者关系、供受者血型、回输单个核细胞和CD34^+^细胞数量、移植前MRD、血液学缓解（HCR）状态、粒细胞及血小板植入、急性GVHD、慢性GVHD、CMV感染、EBV感染纳入单因素分析，结果显示影响生存的危险因素为MRD阳性、血小板未植入、发生急性GVHD（*P*<0.05）。影响复发的危险因素为MRD阳性、发生急性GVHD（*P*<0.05）。将上述影响因素纳入COX多因素分析模型，结果显示移植前MRD阳性、发生急性GVHD是影响生存和复发的的独立危险因素（*P*<0.05），详见表2。

**表2:** 急性髓系白血病患者单倍型造血干细胞移植后生存和复发影响因素的单因素和多因素分析结果

变量	单因素分析			多因素分析		
	*HR*	95％*CI*	*P*值	*HR*	95％*CI*	*P*值
总生存						
移植前MRD（阴性，阳性）	0.309	0.137～0.695	0.005	0.402	0.172～0.938	0.035
血小板植入（否，是）	22.877	8.288～63.144	0.001	9.475	2.902～30.940	0.001
急性GVHD（无，有）	0.240	0.089～0.647	0.005	0.458	0.149～1.407	0.033
复发						
移植前MRD（阴性，阳性）	0.280	0.124～0.631	0.002	0.344	0.154～0.784	0.003
急性GVHD（无，有）	2.297	1.151～4.583	0.018	2.234	1.116～4.470	0.023

## 讨论

本研究结果显示在移植前获得血液形态学完全缓解并接受haplo-HSCT的AML患者中，MRD对移植预后有明显的影响，而移植前血细胞的恢复状态不影响移植预后。

在AML患者中，对诱导化疗的反应是AML患者预后的一个有力的预测指标，然而既往的研究也大多没有包含血细胞的恢复状态。本研究中，将移植前血细胞的恢复状态分为CR和CRp+CRi两组，结果显示对移植后生存和复发没有影响。Khoan等在一项研究中纳入270例行同胞全相合和无关供者移植的AML患者，移植前HCR的恢复程度同OS、LFS无统计学差异[27]。本中心在一项回顾性研究结果中也显示，在同胞相合造血干细胞移植的AML患者中，移植前CRp+CRi状态对OS、LFS、CIR、非复发死亡（NRM）均无影响[28]。Ciftciler等[18]在一项单中心回顾性研究中也发现移植前血细胞恢复程度对allo-HSCT的预后无影响。近期Innes等[19]报告了一项纳入接受allo-HSCT的155例AML患者的研究结果，血液学未完全恢复的HCR对移植后OS、NRM有显著影响（尤其是NRM而非CIR）。本研究结果显示血细胞恢复的状态对预后无影响，同上述研究结果相似，但也存在差异，可能与不同研究之间预处理方案、供者来源、种族人群等差异有关，且本研究为单中心回顾性研究，因此大规模、多中心研究有待开展。

大量的研究显示，移植前MRD阳性是低生存、高复发的危险因素[13],[24]。Buckley等[25]在一项Meta分析中证实移植前MRD阳性的患者生存率降低（59.7％）、复发率升高（37.9％）。虽与我们的研究结果相似，但因为移植预处理方案、供者类型等差别，值得进一步研究。有意思的是，在haplo-HSCT中，本中心既往研究发现移植时MRD阳性对AML患者对移植预后无影响，提示haplo-HSCT可克服MRD阳性预后的不良影响[26]。本研究结果发现MRD阳性患者生存率低（75.8％）、复发率高（25.5％），考虑本研究中77.8％（42/54）的MRD阳性患者为移植前持续性阳性，与刘竞等[15]在145例移植前持续性MRD阳性的AML患者中复发率升高（38.5％）的研究结果相似。这是否说明，haplo-HSCT可以克服移植前单一时间点MRD阳性对移植预后的影响，但对持续性MRD阳性的患者仍不能完全改变预后，因此对MRD持续阳性的患者是否通过强化预处理、移植后预防性DIL、靶向药物等手段提高疗效有待进一步研究。

此外，本研究多因素分析显示，急性GVHD、血小板植入也是影响生存和复发的独立危险因素，同既往研究结果[29]–[30]相符。本研究为单中心回顾性分析、随访时间较短，统计结果可能造成偏倚，需多中心前瞻性研究进一步证实。

本研究结果显示，在接受haplo-HSCT的AML患者中，持续性MRD阳性是影响移植后生存的独立危险因素，而血细胞恢复程度对移植预后无影响，提示移植前获得MRD阴性对移植疗效有重要意义。
